# An Immune-Related Gene Signature for Predicting Neoadjuvant Chemoradiotherapy Efficacy in Rectal Carcinoma

**DOI:** 10.3389/fimmu.2022.784479

**Published:** 2022-05-06

**Authors:** Liwen Qian, Xiaojing Lai, Benxing Gu, Xiaonan Sun

**Affiliations:** Department of Radiation Oncology, Sir Run Run Shaw Hospital, Zhejiang University School of Medicine, Hangzhou, China

**Keywords:** rectal carcinoma, neoadjuvant chemoradiotherapy, immune-related genes, Bioinformatics analysis, prognosis

## Abstract

**Background:**

Locally advanced rectal cancers (LARC) show a highly variable response to neoadjuvant chemoradiotherapy (nCRT), and the impact of the tumor immune response in this process is poorly understood. This study aimed to characterize the immune-related gene expression profiles (GEP), pathways, and cell types associated with response or resistance to neoadjuvant chemoradiotherapy.

**Methods:**

The transcriptomic and clinical data of Rectal carcinoma from the Gene Expression Omnibus database and Immune-related genes (IRGs) from ImmPort were downloaded to identify the differentially expressed immune-related genes (DEIRGs) between responder and non-responder to neoadjuvant chemoradiotherapy. Gene set enrichment analyses were performed to uncover significantly enriched GO terms and KEGG pathways. Immune cell infiltration was estimated from RNA-sequencing data using ImmuCellAI. Afterward, we constructed an immune-related gene-based predictive model (IRGPM) by Support Vector Machine and validated it in an external cohort.

**Result:**

A 15-gene signature (HLA-DPB1, HLA-DQA1, CXCL9, CXCL10, TAP2, INHBB, BMP2, CD74, IL33, CCL11, CXCL11, DEFB1, HLA-DPA1, CCN3, STAT1) was identified as DEIRGs and found to be significantly associated with nCRT outcomes. Gene set enrichment analyses indicated that the 15 genes play active roles in inflammation-related biological processes. In addition, ImmuCellAI revealed that CD4 naive T cells, Tex, Th1 were significantly up-regulated (p=0.035, p=0.02, p=0.0086, respectively), while Tfh were significantly down-regulated (p=0.015) in responder subgroup. Finally, a novel predictive model was developed by SVM based on DEIRGs with an AUC of 80% (internal validation) and 73.5% (external validation).

**Conclusion:**

Our team conducted a genomic study of the relationship between gene expression profile and response to nCRT in LARC. Our data suggested that the DEIRGs signature could help predict the efficacy of nCRT. And a DEIRGs‐based SVM model was developed to monitor the outcomes of nCRT in LARC.

## Introduction

Rectal cancer (RC) is one of the most common malignancies, amounts to one-third of colorectal cancer, which is viewed as the third leading cancer and the second cancer-related mortality in men and women worldwide (1). And the majority of the patients are diagnosed with invasive stage, should be referred for multidisciplinary treatment. For patients with locally advanced rectal cancer (LARC), neoadjuvant chemoradiotherapy (nCRT) followed by radical surgery with total mesorectal excision (TME) has become the standard of care according to the clinical practice guideline of NCCN (National Comprehensive Cancer Network) (2). As was demonstrated in previous research, nCRT has emerged as an indispensable component of curative therapy, which offers a higher probability to downsize and downstage tumors, to enhance tumor resectability and sphincter preservation, and to improve local control rate (3–6). However, LARC exhibits heterogeneity in response to nCRT, varying from nonresponse to complete pathologic response (7). Given the spectrum of response, tailored decisions should be made individually by physicians. However, there is a lack of effective methods to select rectal cancer patients who would or would not have a benefit from nCRT. Numerous efforts have been made, but none has currently reached the clinic (8).

The behavior of a tumor is not only governed by the epithelial component but also by the tumor environment (9). As an essential component of the tumor microenvironment, the tumor immune microenvironment (TIME) serves a vital role in tumor progression and treatment outcome, has gradually acquired accumulative attention. Mounting evidence indicates that characterizing TIME could enable the identification of new prognostic and predictive biomarkers and the possibility to guide first-line treatment algorithms (10). Some features of TIME have been quantified and incorporated into an index called ‘immunoscore,’ which can be used as a strong predictor of patient survival in colorectal carcinoma, providing information more robust than the standard AJCC/UICC-TNM staging system and microsatellite instability (11–14). Therefore, the contexture of TIME has the potential to predict response to nCRT, which has been confirmed in a recent study (15). But the preliminary findings need to be further corroborated in large patient cohorts. Herein, it is urgent to construct an immune-related gene signature closely related to TIME, aiming to predict nCRT efficacy.

This study aimed to characterize the immune-related gene expression profiles (GEP), pathways, and cell types associated with response or resistance to neoadjuvant chemoradiotherapy. Initially, we mainly focused on significant immune genes associated with poor tumor response through data mining in the Gene Expression Omnibus (GEO) databases. We found that differentially expressed immune-related genes (DEIRGs) were significantly associated with the efficacy of nCRT. Then, Gene set enrichment analyses were performed to uncover greatly enriched GO terms and KEGG pathways. Immune cell infiltration was estimated from RNA-sequencing data using ImmuCellAI. Finally, an immune-related gene-based prognostic model (IRGPM) was constructed by Support Vector Machine and validated in an external database.

## Materials and Methods

### Data Acquisition

The publicly available mRNA expression profiles and corresponding clinical information were downloaded from the Gene Expression Omnibus (GEO) database (https://www.ncbi.nlm.nih.gov/geo/), with accession numbers GSE87211, GSE45404, and GSE35452. The gene expression profiles (ID, GSE87211) were previously produced using the Agilent-026652 Whole Human Genome Microarray 4x44K v2 (Probe Name version). Specimens in GSE87211, including 203 tumor samples and 160 mucosa control samples, were obtained from 243 patients. All patients were staged as locally advanced (cUICCII/III/IV) and treated according to the CAO/ARO/AIO-94 or the CAO/ARO/AIO-04 trial (16). The other two microarray datasets (ID, GSE45404, and GSE35452) were previously produced using Affymetrix HGU133 Plus 2.0 GeneChips (Affymetrix, Santa Clara, Ca).

The specimens in the GSE45404 were obtained from 42 patients with LARC (91% and 88% of patients were clinically staged as T3–4 and lymph nodes positive, respectively). 38 (90%) patients received a total dose of radiotherapy higher than 50 Gy, and 15 out of these cases (36%), drugs other than 5-FU were administered (n=11, Oxaliplatin; n=4, Carboplatin). For 33 (79%) patients, 5-FU was administered by continuous venous infusion. The median (range) interval time between the completion of nCRT and surgery was 46 (30–66) days. The tumor response to nCRT was defined as the tumor regression grade (TRG) and was scored following the criteria proposed by Mandard et al. (17).

The specimens in the GSE35452 were obtained from 46 patients with LARC. All patients received a total dose of 50.4Gy of radiation, given in 28 fractions over six weeks. Tegafur-uracil (300-500mg/day) and leucovorin (75mg/day) was given concomitantly with radiotherapy. Standardized curative resection was performed six weeks after the completion of nCRT. Response to nCRT was determined by histopathological examination of surgically resected specimens based on a semiquantitative classification system defined by the Japanese Society for Cancer of the Colon and Rectum (18).

### Screen Genes of Differential Expression

The rectal cancer-related gene expression profile was identified in the GSE87211 database. And GSE45404 database was used to select genes associated with nCRT efficacy by comparing responders and non-responders. We utilized the R package ‘limma’ to screen the differentially expressed genes (19). Those genes with P-value < 0.05 and the expression ratio lg |fold change| > 0.5 were picked out for further evaluation. A Benjamini-Hochberg FDR of 0.1 was used to identify highly significant associations to account for multiple hypothesis testing.

#### Generation of DEIRGs

1793 IRGs were retrieved from 17 categories after excluding the duplicates from the Immunology Database and Analysis Portal (ImmPort) website (https://www.immport.org) (20). The overlapping DEGs in GSE87211 and GSE45404 datasets were intersected with immune-related genes from ImmPort to obtain the target genes defined as the differentially expressed immune-related genes (DEIRGs).

#### Gene Set Enrichment Analysis

Gene set enrichment analysis (GSEA), also called functional enrichment analysis, is a popular calculation method applied to analyze the slight changes in the expressions of genes that belong to a critical pathway. So GSEA can be used to understand the molecular mechanisms of complex disorders, lead to the discovery of new suspect genes and biological pathways, and help provide molecular evidence for the association of biological pathways with diseases (21). In our study, Gene set enrichment analysis was performed and illustrated by ‘Clusterprofiler’ (22) and ‘Pathview’ (23) packages in R. Gene Ontology (GO) classification, including GO-BP (biological process), GO-MF (molecular function), and GO-CC (cellular component), was used to uncover the functions of intersecting genes and further test the biological links in DEGs. GO terms with significant P values were selected according to the BP, CC, and MF results. The pathways with the following criteria were significantly enriched: nominal p-value < 0.05, false discovery rate (FDR) q-value < 0.25.

### Estimation of Immune Cell Infiltration

To explore the associations between nCRT efficacy and immune cell infiltration, we employed Immune Cell Abundance Identifier (ImmuCellAI) (24), a valuable resource for comprehensive analysis of tumor-infiltrating immune cells. ImmuCellAI is a tool to estimate the abundance of 24 immune cells from gene expression datasets, including RNA-Seq and microarray data. It can be applied to estimate the difference in immune cell infiltration in diverse groups. The core algorithm of ImmuCellAI includes three main steps: 1) reference expression matrix and marker gene preparation, 2) enrichment score calculation, and 3) compensation matrix correction. The reference expression profiles of the immune cells were obtained from GEO, and marker genes per immune cell type were obtained from the literature and analytical methods. For each queried sample, the enrichment score of the total expression deviation of the signal gene sets was calculated and assigned to each immune cell type by the ssGSEA algorithm. The compensation matrix and least square regression were implemented to correct the bias caused by the shared marker genes among different immune cell types.

### Construction of an SVM Classifier

Machine learning with maximization (support) of separating margin (vector), called support vector machine (SVM), is a powerful classification tool that has been used for cancer genomic classification or subtyping (25). SVM aims to create a decision boundary between two classes that enables the prediction of labels from one or more feature vectors (26). because our classification problem was nonlinear, the polynomial kernel was adopted, the formula as follows:


$$K(x,z)=(\gamma <x,z>)+c{)}^{d}$$

To evaluate the predictive accuracy of DEIRGs for “N” and “NR” samples, a supervised SVM classifier was constructed and trained *via* the SVM function in the ‘e1071’ package of the R software.

### Validation Analysis

The efficacy of the SVM model was further verified in the GSE45404 dataset (internal validation set) and GSE35452 dataset (external validation set), respectively. Receiver operating characteristic (ROC) curves were used to evaluate the sensitivity and specificity of the predicting model by the ‘pROC’ package in R. The area under the curve (AUC) ≥ 0.7 indicated a good differentiation ability. To more detailedly confirm the prediction value of the SVM classifier model, an SVM with a confusion matrix was constructed.

### Statistical Analysis

Most statistical analyses were performed using R software (Version 4.0.2; R Foundation for Statistical Computing, Vienna, Austria). Graphs related to R statistical analyses were drawn using the ggplot2 package in R.

## Result

### An Immune-Related Signature of Chemo-Radio Resistance in LARC

**Figure 1:**
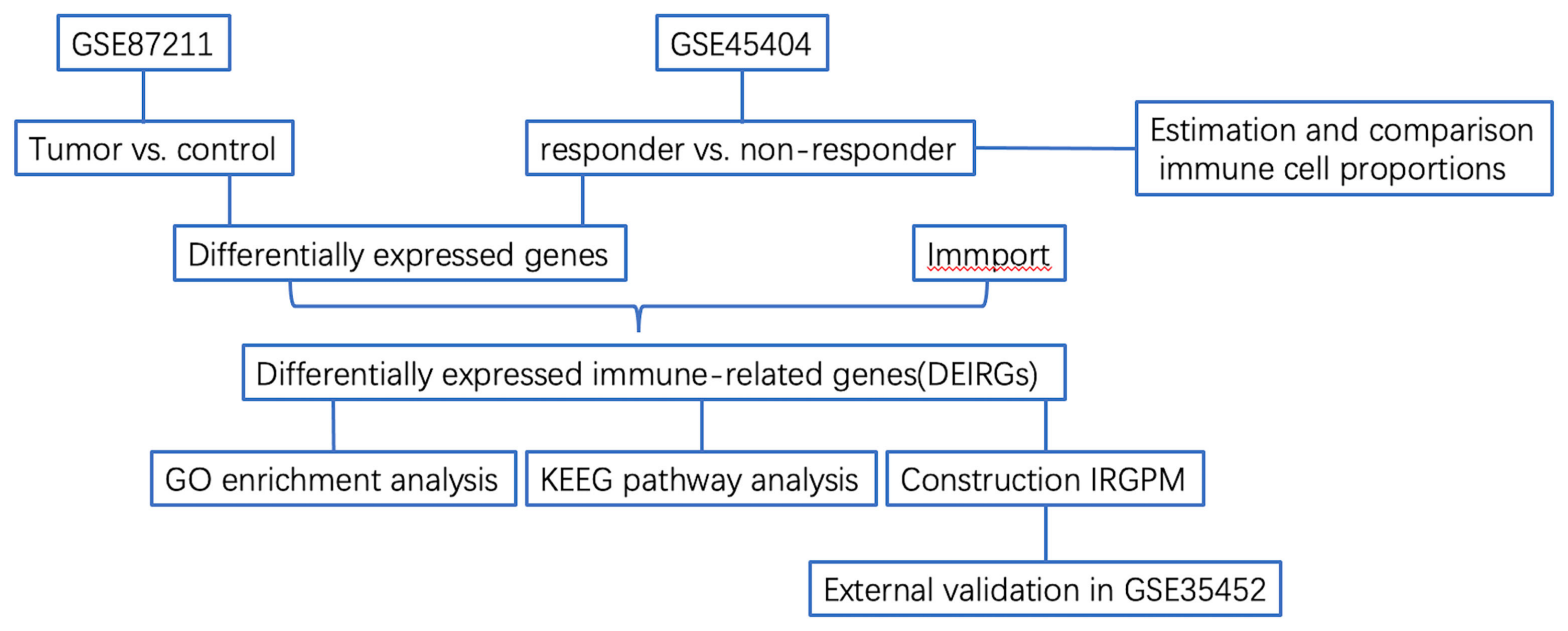
Flowchart describing the process used to identify DEIRGs and construct IRGPM of rectal carcinoma.

**Figure 2:**
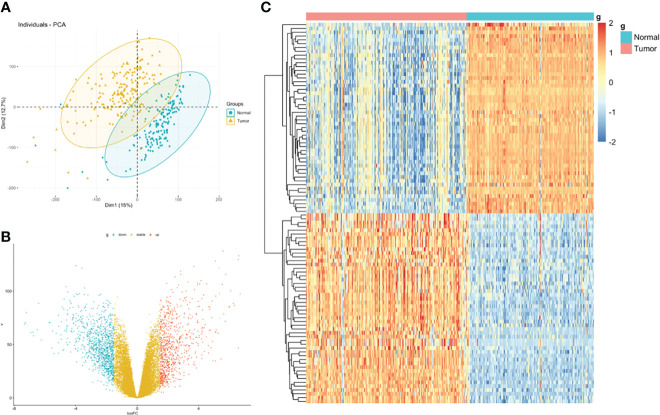
Identification of DEGs between rectal carcinoma tissue and mucosa control tissue. **(A)** Principal component analysis (PCA) of expression level of rectal cancer and normal tissue. **(B)** Volcano plot and **(C)** hot map illustrating differentially expressed genes (DEGs) between rectal carcinoma tissue and mucosa control tissue.

The flowchart of the procedures in this study is shown in **Figure 1**. Three independent datasets were enrolled in our study (**Table 1**). Initially, we focused on gene expression profiles (GEP) of rectal carcinoma to reveal the underlying mechanism of rectal cancer. DEGs analysis was performed between the tumor and normal tissue in the GSE87211 cohort. A total of 7941 deregulated genes were identified, which were defined as RC-related DEGs. A volcano plot and heatmap were generated to show the distribution of the DEGs (**Figures 2B, C**). Besides, the principal component analysis (PCA) was applied to analyze the whole gene expression, and the 2D-PCA plot showed significant differences between rectal cancer and normal tissue (**Figure 2A**).

**Table 1:** Characteristics of three datasets.

Dataset	GSE87211	GSE45404	GSE35452
Status	Public on Nov28 2017	Public on Jan 01 2017	Public on Jan 29 2013
Institution	National Cancer Institute, USA	University of Padova, Italy	University of Tokyo, Japan
Platform	GPL13497	GLP570	GPL570
No. of patients	243	42	46
No. of samples	Tumor:203	R tumor: 19	R tumor: 22
	Mucosa control: 160	NR tumor:23	NR tumor: 24
Data row count	34127	54675	54675

**Figure 3:**
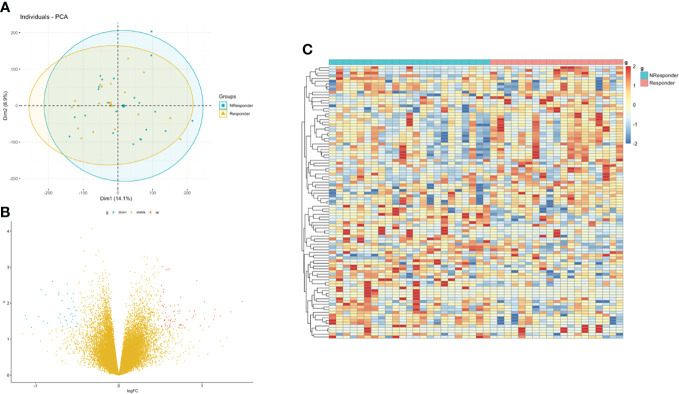
Identification of DEGs between rectal responder (R) subgroup and non-responder (NR) subgroup. **(A)** Principal component analysis (PCA) of expression level of rectal responder (R) subgroup and non-responder (NR). **(B)** Volcano plot and **(C)** hot map illustrating differentially expressed genes (DEGs) between rectal responder (R) subgroup and non-responder (NR).

Then, we integrated gene expression signatures to search for differences in gene expression linked to nCRT response and obtain information on the molecular mechanisms involved in differential nCRT efficacy. DEGs analysis between responders and non-responders in the GSE45404 cohort was performed. A total of 73 upregulated and 40 downregulated genes were identified, defined as nCRT-related DEGs. A volcano plot and heatmap were generated to show the distribution of the DEGs (**Figures 3B, C**). Auxiliary, the principal component analysis (PCA) was applied to analyze the whole gene expression, and the difference between responders and non-responders was not significant in the 2D-PCA plot (**Figure 3A**).

**Figure 4:**
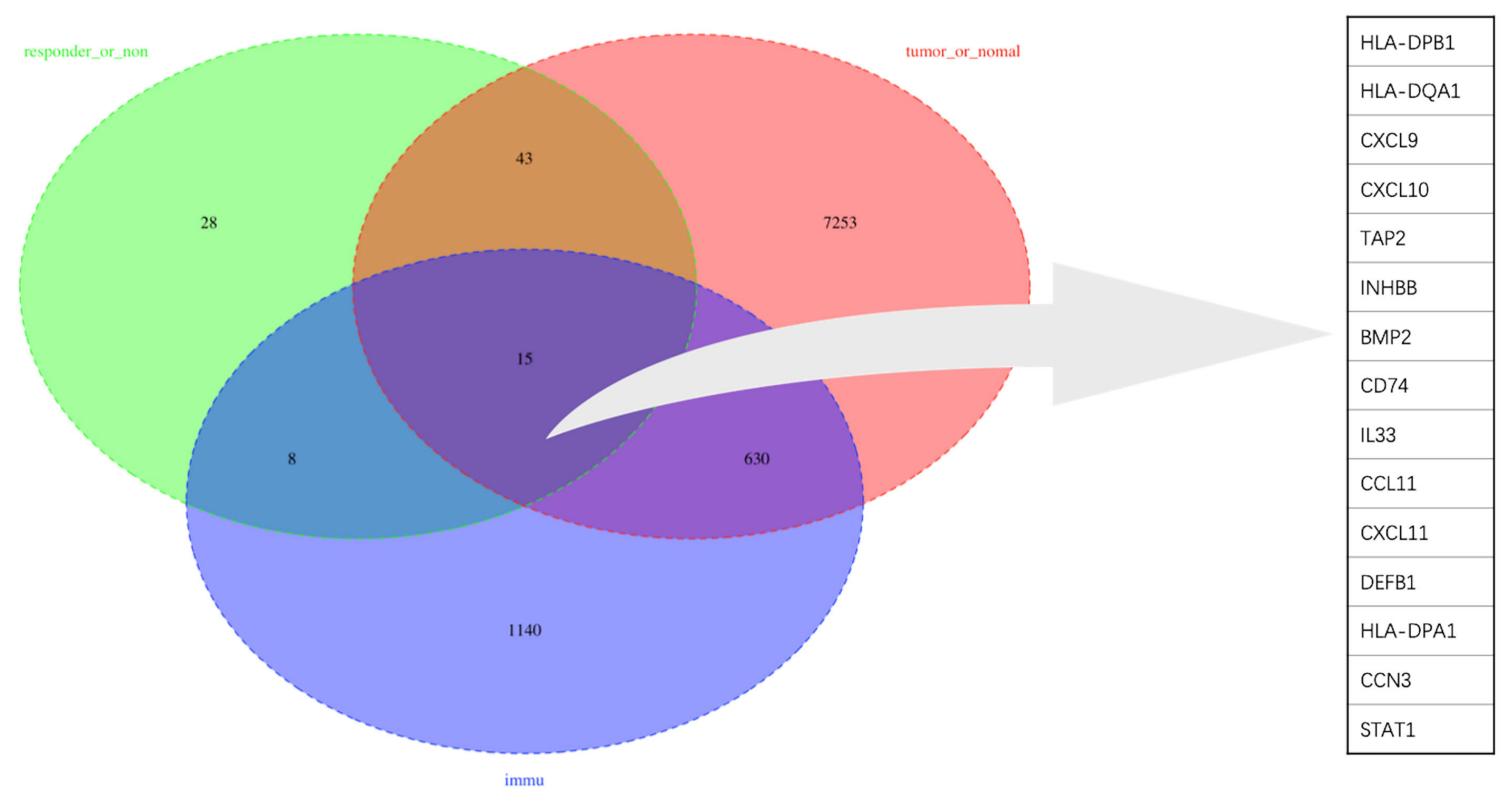
Venn diagram of the overlapped genes between DEGs and IRGs.

To obtain a robust gene signature to predict nCRT efficacy, GO enrichment analysis of biological processes was conducted in the overlapping DEGs in GSE87211 and GSE45404 datasets, which demonstrated “immune-related responses” were the most frequently used biological terms in biological processes (**Supplementary Figure 1**). In this regard, an immune-related gene signature may be able to predict chemo-radio resistance. To address the issue, the 58 overlapping DEGs were intersected with 1793 immune-related genes from ImmPort to obtain the target genes that were defined as the differentially expressed immune-related genes (DEIRGs) (**Figure 4**). In the end, 15 DEIRGs were generated, and the details were shown in **Table 2**.

**Table 2:** The 15 differentially expressed immune-related genes.

Gene	ID	logFC	AveExpr	Deregulation	P.Value	FDR	Description
HLA-DPB1	3115	0.73	8.18	↑R	0.016	0.050	major histocompatibility complex, class II, DP beta 1
HLA-DQA1	3117	1.49	6.30	↑R	0.009	0.050	major histocompatibility complex, class II, DQ alpha 1
CXCL9	4283	1.15	6.13	↑R	0.023	0.050	C-X-C motif chemokine ligand 9
CXCL10	3627	1.16	7.25	↑R	0.015	0.050	C-X-C motif chemokine ligand 10
TAP2	6891	0.54	4.51	↑R	0.004	0.050	transporter 2, ATP binding cassette subfamily B member
INHBB	3625	-0.68	5.38	↓R	0.008	0.050	inhibin subunit beta B
BMP2	650	-0.55	5.88	↓R	0.024	0.050	bone morphogenetic protein 2
CD74	972	0.59	10.61	↑R	0.043	0.055	CD74 molecule
IL33	90863	-1.05	5.05	↓R	0.047	0.052	interleukin 33
CCL11	6356	1.09	7.01	↑R	0.045	0.052	C-C motif chemokine ligand 11
CXCL11	6373	0.98	3.93	↑R	0.042	0.052	C-X-C motif chemokine ligand 11
DEFB1	1672	-0.91	5.39	↓R	0.032	0.050	defensin beta 1
HLA-DPA1	3113	0.90	8.02	↑R	0.023	0.050	major histocompatibility complex, class II, DP alpha 1
CCN3	4856	-0.58	3.21	↓R	0.048	0.056	cellular communication network factor 3
STAT1	6772	0.52	6.44	↑R	0.019	0.050	signal transducer and activator of transcription 1

↑, upregulation; ↓, downregulation.

### GSEA Analysis of DEIRGs

**Figure 5:**
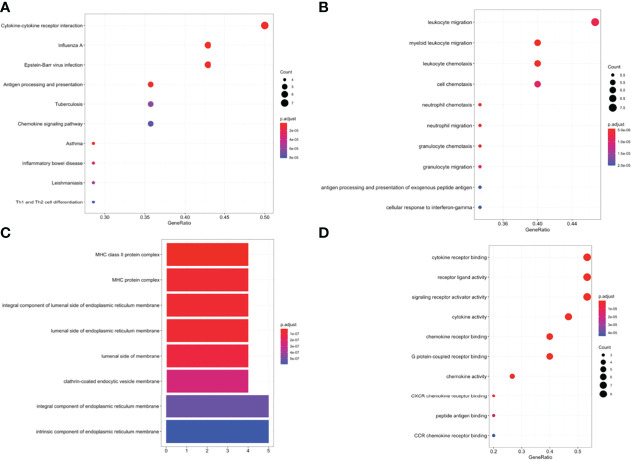
**(A)** Enriched Kyoto Encyclopedia of Genes and Genomes (KEGG) pathways of DEIRGs. Enriched Gene Ontology (GO) pathways of DEIRGs. **(B)** BP biological process, **(C)** CC cell component, **(D)** MF molecular function.

GO, and KEGG functional enrichment analyses were performed on the fifteen selected genes. KEGG pathway analysis showed that the DEIRGs were mainly enriched in cytokine-cytokine receptor interactions (**Figure 5A**). Concerning biological processes, the DEGs were significantly enriched in leukocyte migration, myeloid leukocyte migration, leukocyte chemotaxis, and cell chemotaxis (**Figure 5B**). Meanwhile, the most enriched terms in the aspect of molecular function were cytokine receptor binding, receptor-ligand activity, and signaling receptor activator activity (**Figure 5D**). Enrichment analysis of cellular compartment and the corresponding distributions are shown in **Figure 5C**.

### Immune Cell Infiltration Between Responders and Non-Responders

**Figure 6:**
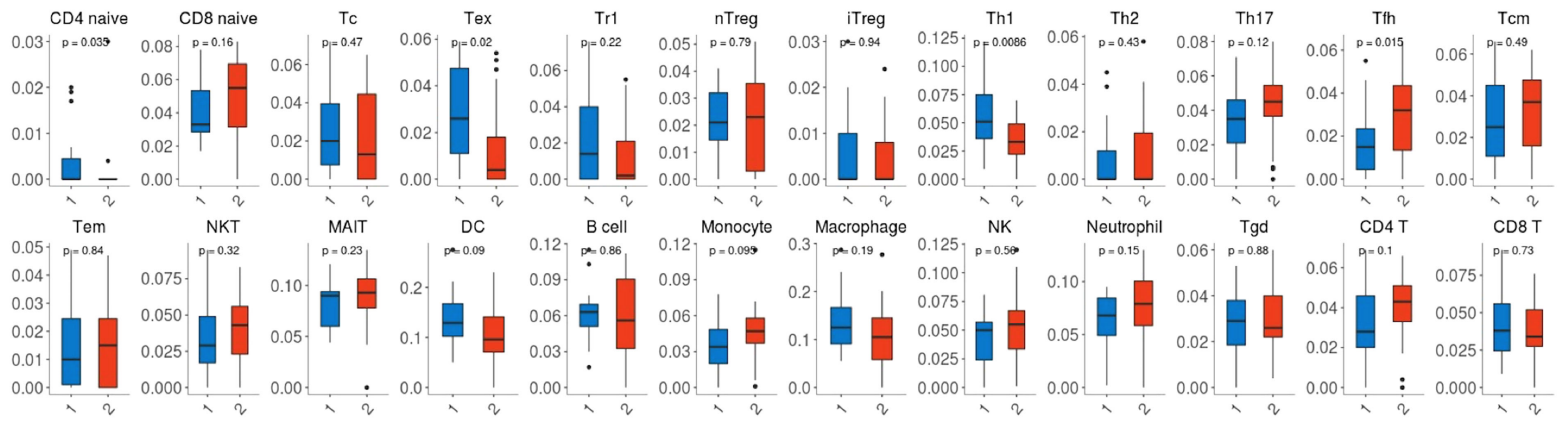
Comparison between the fractions of immune cells in the responder (R) and non-responder (NR) subgroup of the GSE45404 cohort *via* the ImmuCellAI method.

Given that the expression of immune genes was closely associated with nCRT efficacy, it is necessary to investigate the role of TIME on chemo-radio resistance. Twenty-four immune cell fractions were generated using a ssGSEA algorithm to confirm the correlation between TIME and nCRT efficacy. As shown in **Figure 6**, CD4 naive T cells, Tex (exhausted T cells), Th1 (type 1 CD4+ T helper cells) were significantly up-regulated (p=0.035, p=0.02, p=0.0086, respectively), while Tfh (T follicular helper cells) were significantly down-regulated (p=0.015) in responder subgroup of GSE45404 cohort. However, this difference in the distribution ratio of immune cells infiltration was not observed in GSE35452 (**Supplementary Figure 2**).

### Construction of Predictive Model and External Validation

**Figure 7:**
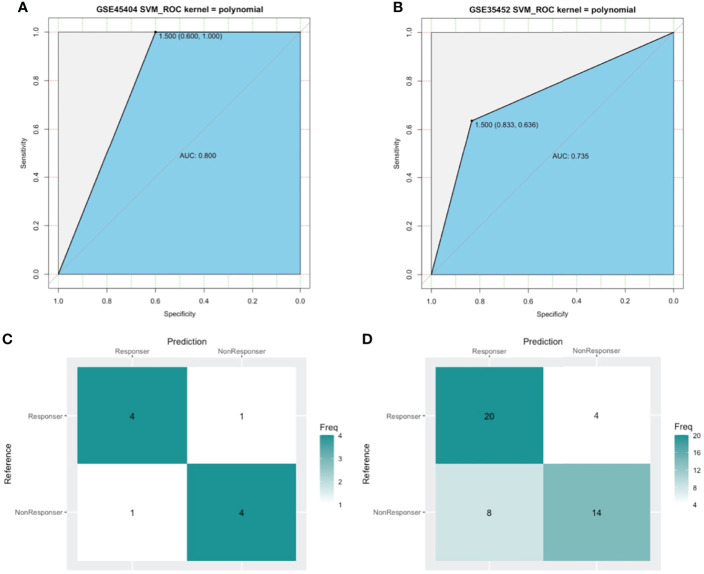
The model measured by receiver-operating characteristic (ROC) curves in GSE45404 dataset (internal validation) and GSE35452 dataset (external validation). **(A, B)** ROC curve. The horizontal axis indicated the specificity and the vertical axis indicated the sensitivity. **(C, D)** confusion matrix. The horizontal axis indicated the predictive case numbers and the vertical axis indicated the present case numbers.

SVM, a method for building a classifier, aims to create a decision boundary, which is orientated in such a way that it is as far as possible from the closest data points from each of the classes. In our study, we addressed the outcome of nCRT as a two-class classification problem (“R” or “NR”) and the above 15 DEIRGs as features. The polynomial kernel was adopted, and the SVM classifier was developed using the GSE45404 dataset. We performed a set of experiments in the training cohort of the first 10 patients, tested the classifier with the remaining 32 samples. As a result, 80% prediction accuracy was found to be achieved using this developed SVM classifier (Kernel=polynomial, cost=1/32, degree=2, gamma =0.125, coef0 = 2) in GSE45404 dataset (**Figure 7A**). Furthermore, SVM with confusion matrix analysis also suggested an 80% prediction accuracy for response samples (sensitivity) and 80% prediction accuracy for nonresponse samples (specificity) (**Figure 7C**).

To further confirm the classification reliability of the above DEIRGs, another microarray dataset GSE35452 was collected and underwent the SVM model analysis. As expected, the SVM exhibited a 73.5% prediction accuracy in the external cohort of 46 samples (**Figure 7B**). The confusion matrix suggested an 83.3% sensitivity, a 63.6% specificity, a 71.4% positive predictive value (PPV), and a 77.8 negative predictive value (NPV) (**Figure 7D**). These findings all proved an outstanding predictive value of this 15 DEIRGs signature.

## Discussion

Response to nCRT varies among patients, and pathological complete response is associated with a better outcome. However, there is a lack of effective methods to select rectal cancer patients who would or would not have a benefit from nCRT. The utility of clinicopathological and radiological features is limited due to a lack of adequate sensitivity and specificity. Although the relationship of gene expression profile and response to nCRT has been well studied (27–29), Predictors of outcome have not been systematically explored in the context of spontaneous tumor immunity. To our knowledge, the predictive ability of the immune gene signature per se is confirmed for the first time.

This study sorted out the most influential immune-related genes affecting patients’ nCRT outcomes with a public database and bioinformatical method. Based on this immune gene signature, we generated an SVM classifier capable of stratifying patients with LARC. Notably, satisfactory AUCs of the SVM classifier imply that the LARC response to nCRT may be affected by the immune system. This conclusion echoes a series of ongoing phase II clinical trials combining nCRT and immunotherapy in LARC patients, such as the VOLTAGE-A trial in Japan, which have achieved better pCR rates (30). Our research found that the DEIRGs and DEIRGs-based SVM model have the potential to be a reference for whether or not to combine immunotherapy in LARC patients.

The immune gene signature consists of 10 upregulated genes and 5 down-regulated genes. The upregulated gene profile consisted of five MHC genes (HLA-DPB1, HLA-DQA1, HLA-DPB1, TAP2, CD74), four chemokine genes (CXCL9, CXCL10, CXCL11, CCL11), and STAT1.

HLA-DPA1, HLA-DQA1, and HLA-DPB1 belong to the HLA class II play a central role in the immune system by presenting peptides derived from extracellular proteins. TAP is essential for peptide delivery from the cytosol to the lumen of the endoplasmic reticulum in the major histocompatibility complex (MHC) class I antigen-presenting pathway (31). TAP expression level in tumor cells defines the nature and processing of MHC class I peptides for recognition by tumor-specific cytotoxic T lymphocytes (32). CD74 associates with class II major histocompatibility complex (MHC) and is a vital chaperone that regulates antigen presentation for immune response. The high expression of these genes indicates an active antigen presentation environment, which is conducive to the presentation of antigens generated during radiotherapy and chemotherapy to tumor killer cells, thereby activating anti-tumor immunity and suggesting better tumor efficacy. Some studies confirmed our conjecture. Restoration of the expression of TAP2 may increase tumor-specific immune responses and survival (33). Expression of CD74 associated with favorable survival for stage III melanoma (34).

CXCL9, CXCL10, CXCL11 are interferon-stimulated chemokines that attract T cells. The chemokine encoded by the CCL11 gene displays the chemotactic activity of eosinophils and shows anti-cancer characteristics in colorectal carcinoma (35). STAT1 signaling promotes T helper 1 (Th1) differentiation and interleukin-12 receptor (IL-12) expression, and thus, in some context, has a role in the anti-tumor immune response (36, 37). Overexpression of these genes may better recruit T cells, thereby activating more robust anti-tumor immune responses.

The five genes downregulated in the R group were involved in tumor progression to varying degrees. INHBB and BMP2 are TGF-β (transforming growth factor-beta) related genes. TGF-β is central to immune suppression within the tumor microenvironment, and recent studies have revealed roles in tumor immune evasion and poor responses to cancer immunotherapy (38). *in vivo* IL33 is involved in the maturation of Th2 cells and activating mast cells, basophils, eosinophils, and natural killer cells. IL33 recruited macrophages into the cancer microenvironment and stimulated them to produce prostaglandin E2, which supported colon cancer stemness and tumor growth (39). CCN3 is a member of the CNN family, which plays crucial roles in CRC progression, including cell migration, invasion, adhesion, and distal metastasis (40). DEFB1 is an antimicrobial peptide implicated in the resistance of epithelial surfaces to microbial colonization, which decreased in colon cancer specimens (41).

Given that Immune-related genes expressed differentially in the subgroups of neoadjuvant radiotherapy and chemotherapy, we suggested that there may be different tumor immune microenvironments between the subgroups. Our data revealed a significant difference in the distribution of some immune cells between the responder group and the non-responder group in GSE45404. In the responder group, CD4+ naive T cells, Th1(type 1 CD4+ T helper cells) increased significantly. It is consistent with the current understanding, as CD4+ naive T cells differentiate into effector T cells (CD4+ helper cells) after stimulation, and Th1 cells produce IL-2 and IFNγ and favor cellular immunity (acting on CD8+ cytotoxic T cell, NK cells, and macrophages). This immune contexture predicts better outcomes in patients with colorectal cancer (42, 43). Previous studies have shown that Pre-nCRT CD8+TILs, CD4+TILs, and MDSC-TILs are sensitive predictors of response to CRT, and high CD8+TILs are associated with a better prognosis (44). We did not observe this phenomenon. In addition, the results of GSE45404 could not be confirmed in GSE35452. Perhaps limited by the limited sample size, the differences observed in GSE45404 are not generalizable. Or different nCRT schemes and different assessment protocols between GSE45404 and GSE35452 databases caused this result. In this regard, we revised the paper.

There were several limitations in our study that should be acknowledged. First, our study was based on publicly available data sets, and it was not possible to obtain complete clinical information (like pCR, recurrence, survival) and demographic data for each patient. Because of insufficient data, our model cannot be used to predict pCR, survival time, and prediction of disease recurrence. Second, as it was a retrospective study, the potential bias relating to unbalanced clinical-pathological features and treatment heterogeneity like not wholly same nCRT scheme and assessment protocol cannot be ignored. Prospective studies are further required to validate the results. Third, among the 15 DEIRG, there were 3 HLA class II genes. These are highly variable genes, and their expression couldn’t be appropriately addressed using microarrays. This factor affected the predictive performance of our model. Forth, Specimens used in this study were obtained from biopsy, and samples from the biopsy could not reflect the tumor because of tumor heterogeneity. Finally, we have not yet added experiments to explore the mechanism behind the immune-related gene signature, and experimental studies on these immune genes are greatly needed. Even so, our findings might provide some reference value for further research in the functional roles of these immune-related genes.

## Conclusion

Overall, our data revealed that the DEIRGs signature could help predict the outcomes of nCRT, and the DEIRGs‐based SVM model could monitor the efficacy of nCRT in LARC. These findings may suggest that immune response plays a vital role in the therapeutic effects of chemoradiation.

## Data Availability Statement

The datasets presented in this study can be found in online repositories. The names of the repository/repositories and accession number(s) can be found in the article/**Supplementary Material**.

## Author Contributions

LQ is mainly responsible for the layout of research ideas, data collection and processing, and the writing of papers. BG and XL participated in data collection and revision of the paperwork. XS made critical revisions to this paper and approved the final version of the article. All authors contributed to the article and approved the submitted version.

## Conflict of Interest

The authors declare that the research was conducted in the absence of any commercial or financial relationships that could be construed as a potential conflict of interest.

## Publisher’s Note

All claims expressed in this article are solely those of the authors and do not necessarily represent those of their affiliated organizations, or those of the publisher, the editors and the reviewers. Any product that may be evaluated in this article, or claim that may be made by its manufacturer, is not guaranteed or endorsed by the publisher.

## Supplementary Material

Supplementary Figure 1 Enriched Gene Ontology (GO) pathways of the overlapping DEGs in GSE87211 and GSE45404 dataset in biological processes.

Supplementary Figure 2 Comparison between the fractions of immune cells in the responder (R) and non-responder (NR) subgroup of the GSE35452 cohort via the ImmuCellAI method.
