# Supplementary figures and images for: An Immune-Related Gene Signature for Predicting Neoadjuvant Chemoradiotherapy Efficacy in Rectal Carcinoma
Source: Front Immunol. 2022 May 6;13:784479. doi: 10.3389/fimmu.2022.784479 (PMC9121132; doi:10.3389/fimmu.2022.784479)

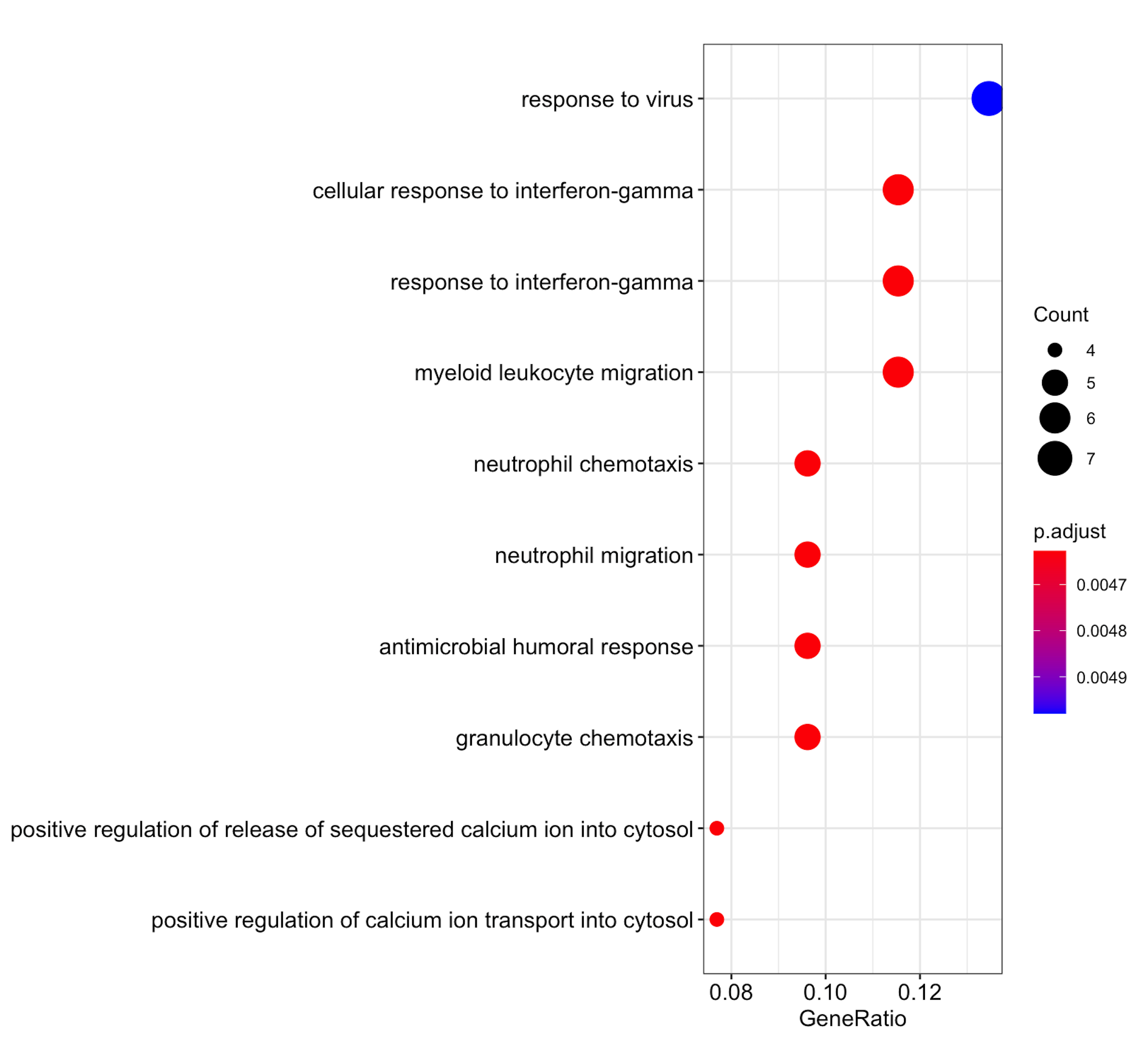

Supplement: Supplementary Figure 1 — Enriched Gene Ontology (GO) pathways of the overlapping DEGs in GSE87211 and GSE45404 dataset in biological processes. [file Image_1.png]

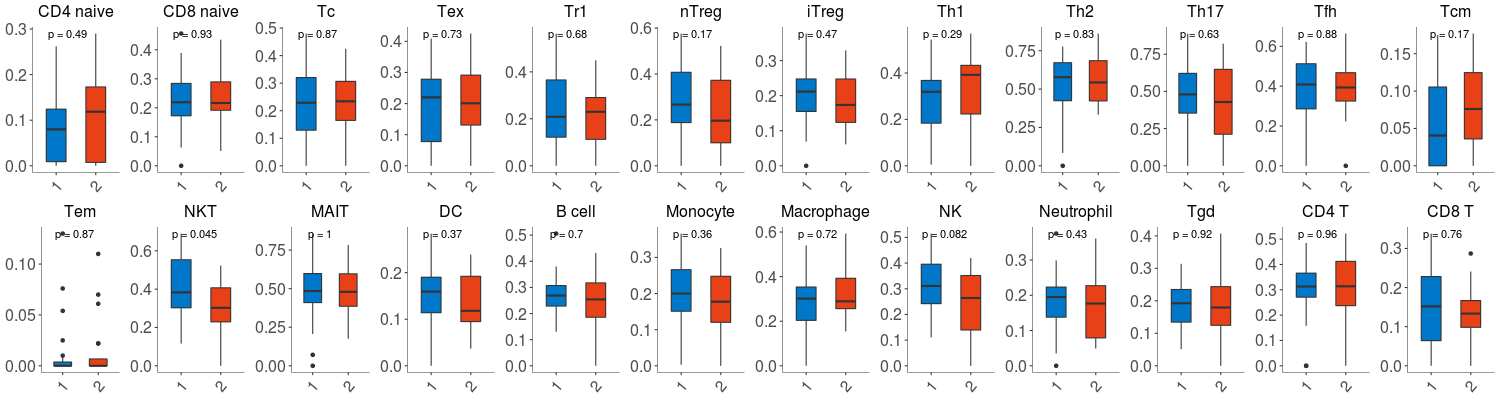

Supplement: Supplementary Figure 2 — Comparison between the fractions of immune cells in the responder (R) and non-responder (NR) subgroup of the GSE35452 cohort via the ImmuCellAI method. [file Image_2.png]
